# Artificial intelligence for rapid on-site evaluation of lymph node fine-needle aspiration: improving diagnostic efficiency and accuracy

**DOI:** 10.3389/fmed.2026.1960803

**Published:** 2026-09-23

**Authors:** Chengcheng Du, Chunhai Li, Hong Meng, Fanlei Kong

**Affiliations:** Department of Radiology, Qilu Hospital of Shandong University, Jinan, China

**Keywords:** artificial intelligence, biopsy, lymph note, rapid on-site evaluation (ROSE), tumor

## Abstract

**Background:**

Lymphadenopathy may result from inflammation, tuberculosis, or tumors, with lymph node status being a key prognostic indicator. Early accurate differentiation of benign and malignant lesions is therefore vital for clinical management. Although imaging offers adjunctive information, histopathology and cytology on biopsy specimens remain the gold standard, albeit with a 2–4 day turnaround. CT-guided biopsy ensures precise sampling, but final diagnosis depends on pathology. AI-ROSE is an emerging real-time cytological tool that can rapidly classify lesions and guide sampling/treatment decisions. While well-validated in lung biopsy, its role in lymph node biopsy is less studied, as most AI research emphasizes imaging over cytology. Therefore, this study assessed the clinical value and diagnostic concordance of AI-ROSE, providing a reference for intraoperative rapid diagnosis and specimen adequacy evaluation.

**Methods:**

This study included 54 patients who underwent lymph node biopsy from June 2024 to June 2026. All samples were obtained by percutaneous puncture under CT guidance and simultaneously underwent AI-ROSE analysis, exfoliative cytology examination, and histopathological examination. Using the histopathological results as the gold standard, the sensitivity, specificity, positive/negative predictive values, and accuracy of AI-ROSE and exfoliative cytology methods were calculated separately, and the consistency between the methods and histopathological diagnosis was analyzed.

**Results:**

The sensitivity of the AI-ROSE diagnosis was 90.48% (95% CI: 77.9% – 96.2%), and the diagnostic accuracy was 89.80% (95% CI: 78.2% – 95.6%). Both of these indicators were higher than those of the exfoliative cytology examination. The consistency between AI-ROSE and the pathological gold standard was moderate (*κ* = 0.647, *P* < 0.001).

**Conclusion:**

In this study, AI-ROSE showed higher sensitivity and diagnostic accuracy than traditional exfoliative cytology for lymph node biopsy. As a practical adjunctive tool, it offers real-time guidance on specimen adequacy and the need for repeat puncture, thereby streamlining the diagnostic process. Notably, AI-ROSE findings should be used as supplementary references and must not replace final histopathological diagnosis.

## 1 Introduction

Lymph node biopsy is applicable to patients with suspected malignant tumors or those with unexplained lymph node enlargement (1). Lymph node enlargement is mainly characterized by an increase in volume and abnormal structure. Some patients may not have a significant increase in the volume of their lymph nodes, but the number may be abnormally increased, and they are still classified as having lymph node lesions. Lymph node enlargement often serves as the initial symptom of various diseases, including hematological disorders. For patients with a high suspicion of malignant tumors or lymph node lesions, lymph node biopsy is the gold standard for clinical diagnosis and the core method for evaluating lymph node diseases (2). Imaging and laboratory tests play a significant role in the screening of lymph node diseases, but they have inherent limitations and cannot replace biopsy. Traditional pathological testing takes 3 to 4 days from sample submission to report issuance, and cytological examination of exfoliated cells also requires 2 to 3 days. This not only prolongs the hospital stay of patients and increases medical costs, but also delays the clinical diagnosis and treatment.

Currently, ultrasound and CT-guided biopsy procedures, due to their minimally invasive nature, low complication rates, and reliable diagnostic results, have become the preferred method for obtaining lymph node tissue samples. These procedures are mainly divided into three types: fine needle aspiration biopsy, hollow needle biopsy, and excisional biopsy. The mediastinal and deep abdominal lymph nodes are located in hidden areas and are difficult to be reached on the surface of the body. For such lesions, fine needle biopsy can replace surgical excision biopsy. This minimally invasive approach can reduce the occurrence of complications and shorten the hospital stay of patients, presenting significant clinical advantages (3, 4). In recent years, with the continuous development of puncture biopsy, histopathology and immunohistochemistry techniques, the application value of percutaneous biopsy in the diagnosis and treatment of lymphoproliferative diseases has become increasingly prominent (5). Rapid On-site Evaluation (ROSE), as an in-surgery real-time supporting technology, can quickly analyze the cellular morphological characteristics of biopsy samples and immediately determine the benign or malignant nature of the lesion (6). With the widespread application of artificial intelligence technology in clinical medicine and pathological diagnosis, the combination of AI and ROSE and its application in interventional examinations and tumor diagnosis have demonstrated great potential for development (7, 9). It can effectively solve the problem of delayed diagnosis caused by the scarcity of pathologists. With this technology, interventional physicians can quickly formulate the next treatment plan after sample collection, significantly reducing the waiting time for patients and helping to achieve precise and timely treatment, thereby improving the overall diagnostic efficiency. This study is based on CT-guided percutaneous lymph node biopsy to analyze the clinical application value of AI-ROSE and verify its diagnostic accuracy and promotion feasibility.

## 2 Methods

### 2.1 Research subjects

A total of 54 patients who underwent lymph node biopsy in Qilu Hospital of Shandong University from June 2024 to June 2026 were included in this study. All cases had definite pathological diagnosis and molecular cytological diagnosis. All patients signed informed consent, and this study was approved by the Medical Ethics Committee of Qilu Hospital of Shandong University (approval number: KYLL-2026-07(YJ)-060). Inclusion criteria included: (1) adult male or female, aged between 18 and 80 years; (2) complete imaging data, abdominal CT or PET-CT imaging suggested lymph node lesions, or clinical highly suspected lymph node neoplastic lesions; (3) stopping anticoagulant and antiplatelet drugs one week before operation; (4) Electrocardiogram, blood routine, coagulation function, infectious disease screening and other preoperative examinations were completed before operation. Exclusion criteria included: (1) patients could not tolerate whole-course lymph node biopsy in general; (2) Severe necrosis of the biopsy specimen; (3) bleeding tendency or coagulation dysfunction; (4) AI-ROSE results were unsatisfactory (The experimental design is shown in Figure 1).

**Figure 1:**
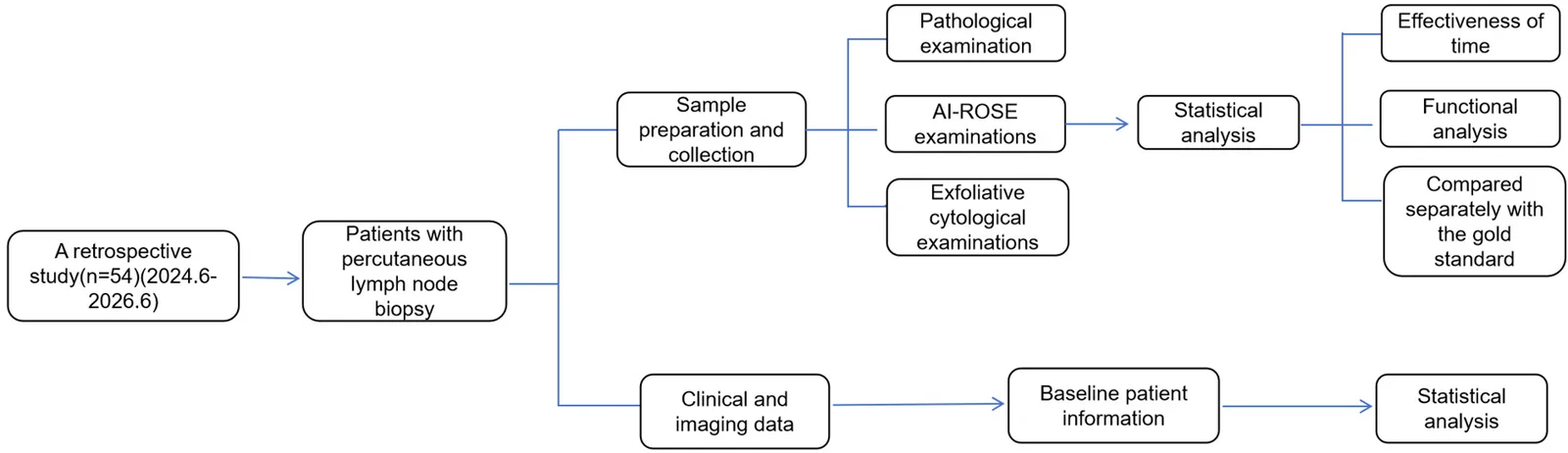
The flowchart of this research.

### 2.2 Study methods and sample

All patients underwent CT-guided percutaneous lymph node biopsy. Patients were placed in the supine or lateral position, and the skin of the puncture area was routinely disinfected, and the anesthetic skin and subcutaneous tissue were locally infiltrated. Under the real-time guidance of CT, the special puncture needle was used to locate the lesion and quickly complete the puncture sampling. The procedure is minimally invasive and safe, which can ensure the accuracy of puncture. After the sampling was completed, tissue samples were smetted, and the remaining tissue was fixed in formalin solution and sent to the pathology department for subsequent analysis (9).

The tissue samples obtained by puncture were about 1 cm in length and 0.1 cm in diameter. The samples were evenly spread on a sterile slide to make a cell smear with a diameter of about 1 cm, and the sample contamination was strictly avoided during the operation. Immediately after the smear, the slides were stained for ROSE assessment by an interventionist. The smear was immersed in solution A for 30–50s by Deave rapid staining method. After removing the smear from solution A, the smear was rinsed in PBS buffer for 4–5 times to remove the staining solution and dirt. Then the slide was immersed in solution B for 20∼40s, and the smear was removed from the B staining solution and rinsed in PBS buffer for 4∼5 times to remove the staining solution and dirt. The slide was lifted up and down several times during the staining process so that the staining was evenly distributed. After the staining was completed, the excess water was blotted with absorbent paper to make the glass slide free of water stains as far as possible, and then the slide was naturally dried and placed in the digital microscopic image analysis system for observation. After instrumental determination, the patient was immediately transported to the cell room for independent evaluation by a cytopathologist who was unaware of the AI results (The examination results are shown in Figure 2). All biopsy procedures and section preparation were performed by interventional physicians with more than 10 years of experience. The experimental materials, equipment, and reagents are detailed in Table 1.

**Figure 2:**
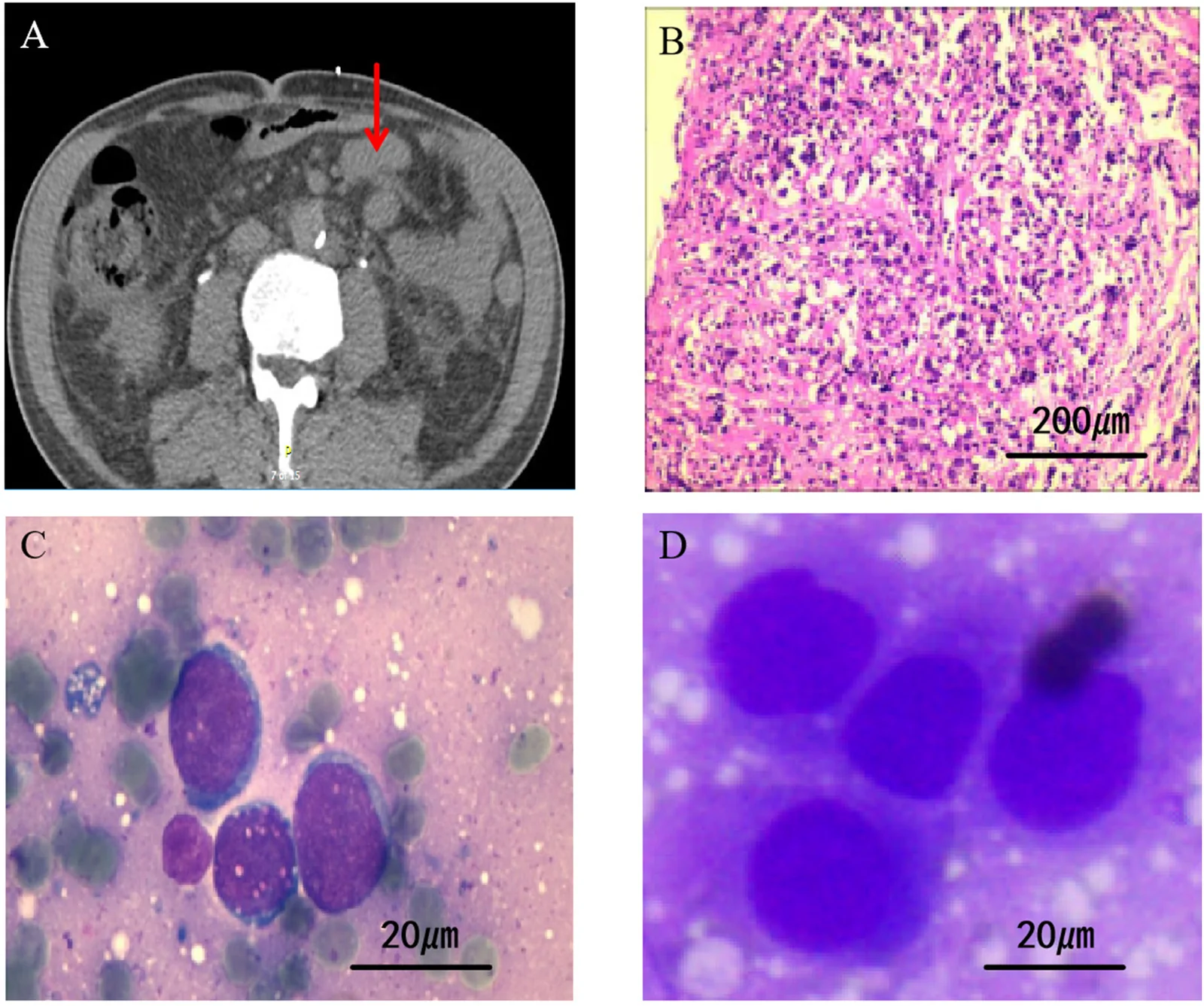
A 67-year-old male patient underwent CT-guided percutaneous needle biopsy of a lymph node. **(A)** The CT image showed a lesion in the abdominal cavity with a longest diameter of approximately 35.5 mm (red arrow); **(B)** histopathological examination suggested diffuse large B-cell lymphoma (× 100); **(C)** cytological examination revealed suspicious malignant cells (×400); and **(D)** the AI-ROSE (Artificial Intelligence Rapid On-Site Evaluation) image showed suspicious malignant cells (×400).

**Table 1:** Experimental materials, equipment, and reagents.

Experimental Materials and Equipment	Model (Specification)	Manufacturer
Digital Microscopy Image Analysis System	AT1	Fosun Xingmai（China）
Diff-Quik Staining Solution A	BA-4100	BASO（China）
Diff-Quik Staining Solution B	BA-4100	BASO（China）
Phosphate buffered solution	500 mL	BASO（China）
ROSE Customized Microscope Slides	26mm*76mm	Fosun Xingmai（China）

### 2.3 Principle of AI-ROSE

In this study, the artificial intelligence cytopathological diagnosis system developed by Shanghai Xingmai Information Technology Co., Ltd. was used to intelligently analyze the digital ROSE cytology slide images. The AI model adopts a two-stage progressive architecture with a multi-step attention enhancement mechanism. The front-end object detection module and the back-end classification module cooperate to analyze the whole cytological image.

For large-size full-slice images, the system crop the original image into standard image blocks of 1024 × 1024 pixels through a sliding window, and input the detection model in batch. The target detection module takes multi-path feature fusion network as the core, selects ViT-B/16 as the feature extraction backbone network, and combines MoCoV2 self-supervised learning strategy for pre-training, which effectively solve the problem of insufficient cytological labeling data, accurately locate and identify abnormal cells, and distinguish normal tissues from pathological tissues. And output the coordinate range of abnormal cells.

According to the detection results, the suspicious lesion area was extracted, and an integrated image of 2048 × 2048 pixels was generated by image splicing, which was used as the input data of the classification module. The lightweight ResNet50 network was used to extract features in the classification module, and the multi-layer perceptron (MLP) was used to complete the binary diagnosis of benign and malignant (as shown in Figure 3).

**Figure 3:**
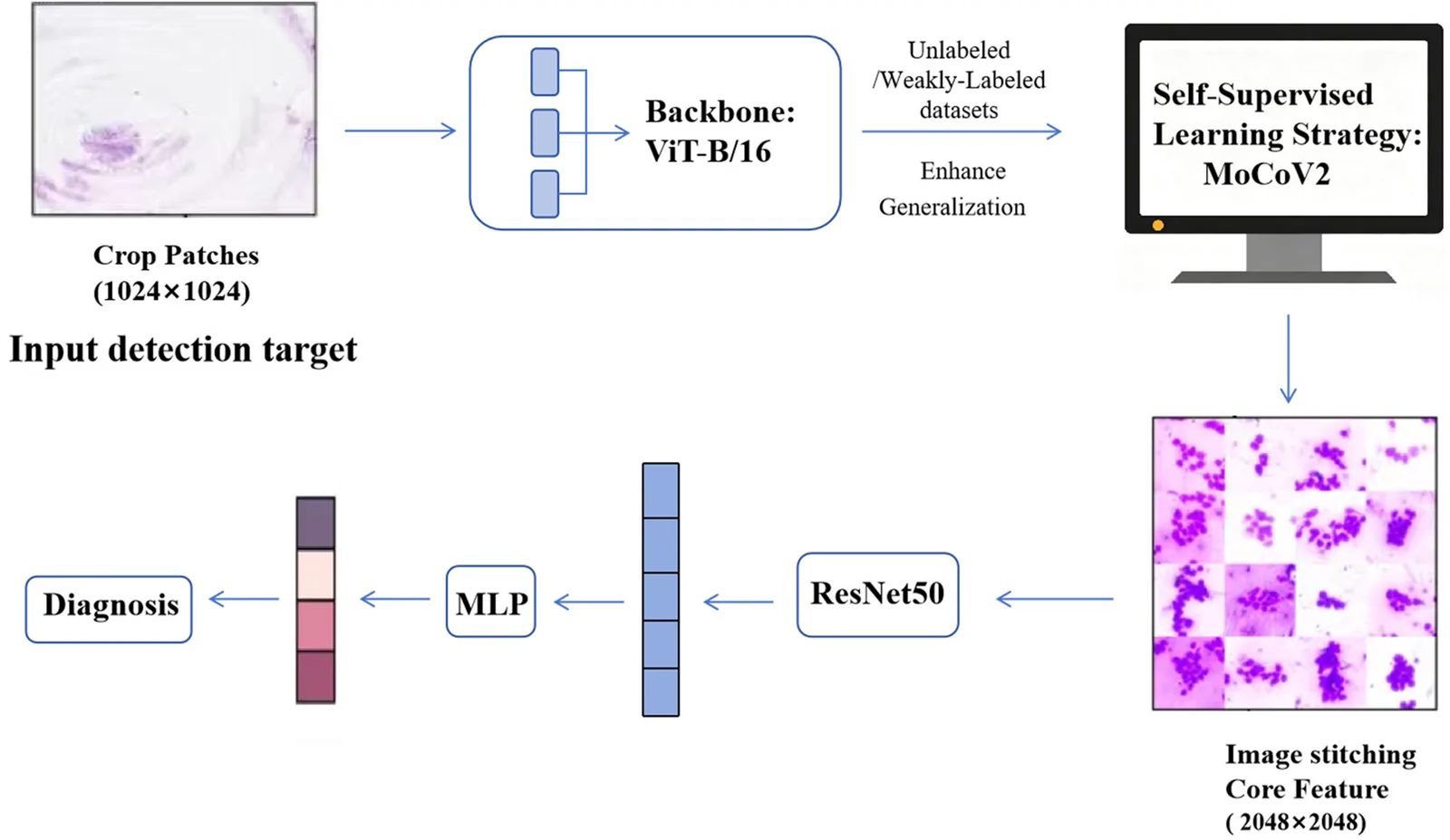
The principle of AI-ROSE: A two-stage progressive architecture with an embedded multi-step attention enhancement mechanism.

The model constructed a tumor cell detection algorithm based on deep learning, and fused SSD algorithm and DenseNet dense connection idea to strengthen the learning of deep features by convolutional neural network. The synchronous batch normalization algorithm was introduced to reduce the risk of overfitting, and Adam algorithm was used to complete the model training after comparing various optimization algorithms. The system assigned the malignant probability of a single image block, and determined benign as a boundary of 0.2 and malignant as a boundary of 0.8. The final diagnosis was made based on the results of the whole image (as shown in Figure 4).

**Figure 4:**
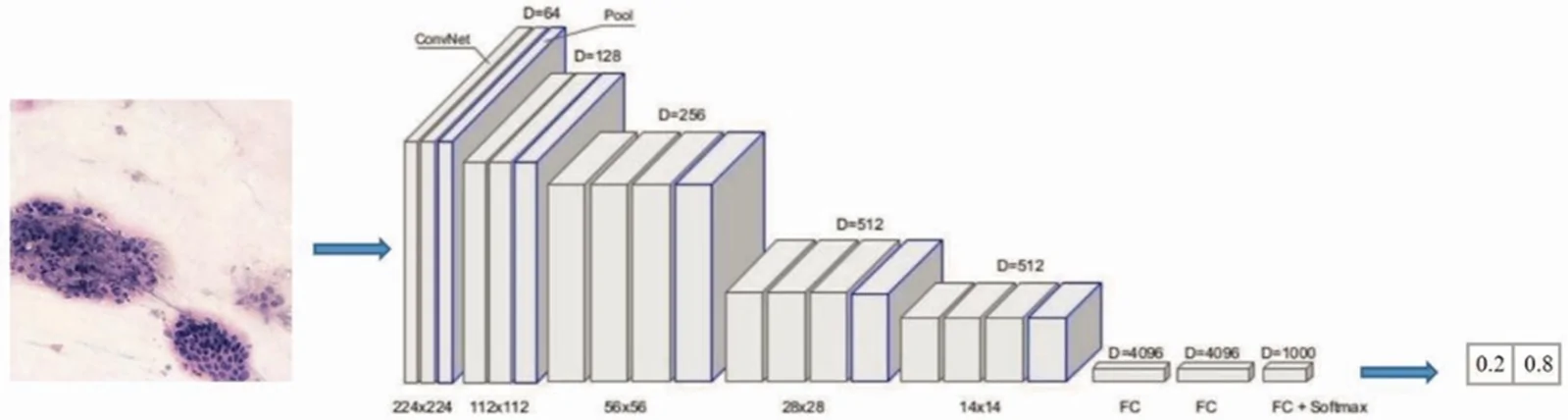
A tumor cell detection algorithm.the system calculates a malignancy probability for each image block. A block is classified as benign if its probability is below 0.2, and as malignant if above 0.8. The final diagnosis is determined by integrating the results across the entire image.

### 2.4 Quality control

Smear preparation: Repeated smear was avoided for a single smear to prevent cell stacking, and unidirectional and gentle smear was used to ensure uniform distribution of cell monolayers and clear and identifiable cell morphology.

Staining time: according to the cell density of the smear, the staining time was adjusted flexibly. The staining time was shortened appropriately for the cell dense smear and extended appropriately for the cell sparse smear to avoid too deep or light staining and ensure clear nuclear and plasma contrast.

Selection of AI reading area: Before automatic AI interpretation, the high-quality area with complete cell morphology, clear staining and no overlapping impurities was manually screened. The AI sampling points in this area were increased to improve the representability and accuracy of diagnosis.

Multi-point sampling review: at least 2 cell smears were routinely prepared for a single puncture. If the amount of cells in a single smear was insufficient and the quality of a smear was poor, the other smears could be used for supplementary diagnosis. The results of multiple smears confirmed each other, which reduced the risk of false negative and the influence of sampling error on diagnosis.

### 2.5 Statistical methods

SPSS 25.0 software was used for data analysis. Statistical content included baseline data analysis, comparison analysis between the two detection methods and pathological gold standard, and subgroup analysis of malignant lesions. The sensitivity, specificity, positive predictive value, negative predictive value and diagnostic accuracy were calculated to evaluate the diagnostic efficacy. Kappa test was used to analyze the diagnostic consistency (*κ* > 0.8 was considered as excellent agreement, 0.6–0.8 as high agreement, 0.4–0.6 as moderate agreement, and < 0.4 as poor agreement). McNemar test was used to analyze the difference of diagnostic results. *P* < 0.05 was considered statistically significant.

## 3 Study results

### 3.1 General information

A total of 54 lymph node tissue samples were collected in this study, including 36 males and 18 females with an average age of 42.57 ± 15.68 years; the demographic characteristics are shown in Table 2. According to AI-ROSE, 10 cases were benign, 39 cases were malignant, and 5 cases were unsatisfactory. Cytology was negative in 24 cases and positive in 30 cases. Pathological results showed that 7 cases were benign and 47 cases were malignant, including 19 cases of primary lesions and 28 cases of metastatic lesions. The characteristics of the patients at baseline are shown in Table 2.

**Table 2:** Demographic characteristics of 54 patients.

Variable	No. of patients (%)
Age (years).mean ± SD	42.57 ± 15.68
Size of lesions(cm).mean ± SD	2.87 ± 1.12
Sex	
Male	36 (66.67%)
Female	18 (33.33%)
Location	
Superficial lymph node	8 (18.97%)
Deep lymph node	46 (81.03%)
Pathological diagnosis	
Benign lesion	7 (12.96%)
Necrotic tissue	2 (28.57%)
Inflammatory lesion	5 (71.43%)
Malignant lesion	47 (87.04%)
Primary lesion	19 (40.43%)
Metastatic lesion	28 (59.57%)
Abnormal serum tumor markers	35 (64.81%)
History of smoking	20 (37.04%)
Drinking history	24 (44.44%)

### 3.2 AI-ROSE interpretation results and consistency with pathological biopsy

Fifty-four patients were found after AI-ROSE dissection, 39 cases were positive, 10 cases were negative, and 5 cases were dissatisfied. The pathological results showed that 7 cases were benign and 47 cases were malignant. Compared with the gold standard of pathological diagnosis, AI ROSE had an accuracy of 89.80%(95%CI: 78.2%–95.6%), a sensitivity of 90.48% (95%CI: 77.9%–96.2%), and a specificity of 85.71%(95%CI: 77.9%–96.2%) in diagnosing lymph node lesions. 48.7%–97.4%), the positive predictive value was 97.44%, the negative predictive value was 60.00%, and the Youden index was 0.7619. The McNemar test showed that there was no significant bias between the two diagnostic results (*χ*^2^ = 0.8, *P* = 0.371). *κ*=0.647 (*P* < 0.001), indicating that AI-ROSE had moderate to good consistency with histopathological results.

### 3.3 Consistency between exfoliative cytology and pathological biopsy

There were 24 negative cases and 30 positive cases in exfoliative cytology. The pathological results showed that 7 cases were benign and 47 cases were malignant. Compared with the gold standard of pathological diagnosis, 29 cases were true positive (TP), 1 case was false positive (FP), 6 cases were true negative (TN), and 18 cases were false negative (FN). The accuracy of cytology in the diagnosis of lymph node lesions was 64.81% (95%CI: 51.5%–76.2%), the sensitivity was 61.70% (95%CI: 47.4%–74.2%), and the specificity was 85.71% (95%CI: 48.7%–97.4%). The positive predictive value was 96.67%, the negative predictive value was 25.00%, and the Youden index was 0.474. Fisher test showed that there was a statistically significant correlation between exfoliative cytology and pathological results (*P* = 0.012), but *κ*=0.233, the consistency was poor. The missed diagnosis rate of this method was high, 18 cases of malignant lesions were misjudged as negative, and the negative predictive value was only 25.00%. McNemar test confirmed that the positive rate of exfoliative cytology was significantly lower than that of pathological diagnosis.

### 3.4 Consistency between AI-ROSE and exfoliative cytology

After excluding 5 cases of unsatisfactory specimens by AI-ROSE, a total of 49 samples were included in the comparative analysis. The diagnostic results of the two methods were the same in 38 cases (the same positive in 29 cases and the same negative in 9 cases). One case was negative by AI-ROSE and positive by cytology. 10 cases were positive by AI-ROSE and negative by cytology. The overall consistency rate was 77.55%, *κ*=0.482 (*P* < 0.001, moderate consistency), Phi = 0.532 (moderate positive correlation). The consistency of positive results was 84.06%, and the consistency of negative results was only 62.07%. AC1 = 0.615, indicating good overall consistency. It can be seen that the two methods have a high fit in the positive interpretation of malignant lesions, and the negative interpretation results are significantly different.

### 3.5 Comparison of AI-ROSE and exfoliative cytology in the diagnosis of lymph node malignancy

Among the 47 patients with pathologically confirmed malignant lesions, 5 patients had unsatisfactory AI ROSE results, and finally 42 patients were included in the valid paired analysis. After AI-ROSE examination, 38 cases were positive and 4 cases were negative. The exfoliative cytology showed 29 positive cases and 13 negative cases. A total of 31 cases were diagnosed by AI-ROSE and exfoliative cytology, including 28 cases with positive diagnosis and 3 cases with negative diagnosis. In addition, one patient was negative by AI-ROSE and positive by exfoliative cytology. Ten cases were positive by AI-ROSE and negative by exfoliative cytology. The positive detection rate of AI-ROSE was 90.5% (38/42), and that of cytology was 69.0% (29/42). McNemar's exact test showed that the detection rate of malignant lesions of AI-ROSE was significantly higher than that of cytology (*P* = 0.012). The total diagnostic consistency rate of the two methods was 73.8%, *κ*=0.24, the consistency was poor. The positive conditional agreement rate was 96.6%, and the negative conditional agreement rate was only 23.1%. The results show that AI-ROSE is more sensitive in the detection of malignant cells, but it still needs to be vigilant against false positive results in clinical application combined with pathological results.

A total of 54 patients with lymph node biopsy were included in this study. With pathological examination as the gold standard, the diagnostic efficacy of AI-ROSE and exfoliated cytology were compared. The time required to obtain results for each method was also assessed. The results showed that the sensitivity and specificity of AI diagnosis were due to traditional exfoliative cytology. There was a significant correlation between AI interpretation and cell results (phi =0.532, *p* < 0.01). There was a moderate positive correlation between AI results and cell results, and the chi-square test (*p* < 0.01) showed that this correlation was statistically significant and not caused by random. In this study, AI-ROSE took an average of 235.60 ± 13.88 s, histopathology 2.27 ± 1.42 days, and cytology 1.28 ± 0.56 days.

## 4 Discussion

In this study, we evaluated the diagnostic performance of AI-assisted rapid on-site evaluation (AI-ROSE) in CT-guided lymph node biopsy against histopathological diagnosis as the gold standard, and compared it with conventional exfoliative cytology. As the gold standard for characterizing nodal disease, biopsy can accurately distinguish reactive hyperplasia and specific infections from malignancies, thereby avoiding misdiagnosis or delay in treatment (10). Our results suggested that AI-ROSE achieved higher accuracy (89.80%) and sensitivity (90.48%) than exfoliative cytology (accuracy 64.81%, sensitivity 61.70%), with moderate-to-good agreement with the pathological reference (*κ*=0.647, *P* < 0.001). These findings appear to support the potential of AI-ROSE as a stable and reliable tool for discriminating between benign and malignant lymph node lesions during biopsy. By obtaining complete pathological tissue, biopsy not only allows for the identification of benign or malignant disease but also permits precise classification and immunohistochemical analysis, which may directly guide subsequent individualized treatment decisions, such as the choice of chemotherapy, targeted therapy, or the extent of surgical dissection (10–14). In cases of persistent lymphadenopathy of unknown etiology and systemic disease, biopsy is considered the most reliable reference for clinical decision making and is likely the key link between diagnosis and clinical management (15).

The apparent superiority of AI-ROSE over traditional cytology may be attributable to the inherent limitations of the latter. Lymphoma is a malignant tumor arising from lymphocytes and lymphoid tissues, and its incidence has increased significantly in recent years (16, 17); common treatment options include surgery, chemotherapy, and radiotherapy (18), many of which rely on pathology and immunohistochemistry. Conventional exfoliative cytology, though operationally simple, relies solely on cellular morphology and fails to preserve tissue architecture—a critical determinant in the diagnosis of lymphomas and metastatic nodal tumours, both of which depend heavily on cellular arrangement and the microenvironment. This fundamental drawback could explain the 18 false-negative cases observed in our cytology group, which yielded a negative predictive value of only 25.00% and poor concordance with pathological diagnosis (*κ*=0.233). In contrast, AI-ROSE employs deep-learning algorithms to extract subtle morphological features, which may reduce the subjectivity of manual reading and might lower the risk of missing malignant lesions. Indeed, the positive detection rate of AI-ROSE for malignant lesions was 90.5%, markedly higher than that of exfoliative cytology (69.0%, *P* = 0.012), and the positive agreement between the two methods reached 96.6%, whereas negative agreement was only 23.1%. This asymmetry seems to further underscore the superior sensitivity of AI-ROSE in detecting malignant cells, potentially compensating for the frequent underdiagnosis by conventional cytology.

When analysing the 49 valid cases with adequate specimen quality, the overall observed agreement between AI-ROSE and exfoliative cytology was 77.55% (*κ*=0.482), indicating moderate consistency, with the AC1 index (0.615) providing a more robust estimate given the unbalanced marginal distributions (positive rate 79.6% for AI-ROSE vs. 61.22% for cytology). Notably, the agreement for positive interpretations was as high as 84.06%, whereas that for negative interpretations was only 62.07%. This disparity suggests that while the positive conclusions from both methods are largely corroborative, negative results should probably not be accepted in isolation; they may need to be integrated with other clinical and laboratory parameters. Therefore, AI-ROSE might be best positioned as a rapid primary screening aid—its positive findings could be highly suggestive of malignancy, but definitive diagnosis would still rely on histopathology and immunohistochemistry.

The clinical value of AI-ROSE may lie primarily in its ability to provide real-time feedback during the procedure, allowing interventionalists to promptly assess specimen adequacy and adjust puncture strategies, thereby possibly avoiding repeated passes due to insufficient material. Compared with the prolonged waiting times for conventional pathology (2–5 days) and exfoliative cytology (1–3 days), AI-ROSE could deliver immediate results, potentially accelerating patient triage, shortening hospital stays, and reducing overall costs (19, 20)—which might be particularly beneficial for patients with high clinical suspicion of malignancy who require urgent therapeutic planning. Rapid on-site evaluation (ROSE) is an important technology in interventional puncture, and AI-ROSE combines artificial intelligence image analysis with ROSE to automate and intelligentize the reading of cytologic samples, with the potential to overcome both the subjectivity of manual interpretation and the dependence on on-site pathologists (21, 22). Moreover, the specificity of AI-ROSE was equivalent to that of exfoliative cytology (both 85.71%), suggesting equally reliable performance in identifying benign conditions such as reactive hyperplasia and infectious lesions. In settings where on-site pathologist support is unavailable, the automated analytical capability of AI-ROSE (23, 24) may offer a practical solution to resource constraints, facilitating wider adoption of rapid on-site evaluation.

Nevertheless, several technical and practical limitations should be acknowledged. The diagnostic performance of AI-ROSE appears to be highly contingent on smear quality, which may be influenced by fixation, thickness, cellularity, necrotic debris, and operator experience. Although we implemented rigorous quality control measures—uniform unidirectional smearing, dynamic adjustment of Diff-Quik staining, manual selection of high-quality fields, and cross-validation with multiple smears—to minimise technical errors, the generalisability of the algorithm remains suboptimal, as variations in staining protocols and imaging devices could affect recognition accuracy. Although AI-ROSE can be performed by trained interventionalists, operator training is recommended to improve diagnostic yield (25–27). Additionally, AI-ROSE cannot provide architectural information, an intrinsic limitation that might be mitigated by integrating novel cytological techniques such as liquid-based cytology and cell blocks, which may provide more abundant morphological and structural information (28, 29).

This study has several shortcomings. It is a single-centre retrospective investigation with a relatively small sample size (54 cases), which introduces potential selection bias and limits the extrapolation of our conclusions. We did not perform stratified analyses based on puncture number, lesion size, or lesion subtype, nor did we compare different AI models or equipment. Future research might focus on: (1) combining AI-ROSE with advanced cytological preparations (e.g., liquid-based cytology and cell blocks) to enrich morphological information and compensate for the shortcomings of pure cytological detection; (2) continuously iterating the AI model through feature learning and fine-tuning on misdiagnosed cases, while integrating imaging data, clinical history, and laboratory indicators to construct a multimodal AI diagnostic model that could break through the limitation of single image analysis; (3) conducting multicentre, large-scale prospective cohort studies to validate the robustness and generalisability of AI-ROSE in real-world practice across different regions, lesion subtypes, and operator skill levels; and (4) exploring its utility in systemic multi-site, recurrent, or metastatic lymph node lesions to broaden its clinical applications.

In summary, AI-ROSE appears to demonstrate high sensitivity and acceptable accuracy in lymph node biopsy, and it may effectively optimise intraoperative workflow and provide objective, real-time screening information. Its positive results seem to correlate well with those of conventional cytology, but negative findings would warrant cautious interpretation. With strict attention to smear quality and operator training, AI-ROSE may represent a valuable and practical adjunctive tool for interventional lymph node diagnosis and treatment, holding substantial promise for clinical translation. The use of AI-ROSE could potentially optimize the intraoperative process and evaluation strategy, ensuring high sensitivity while taking into account prediction accuracy, and might offer an efficient and practical auxiliary scheme for the interventional diagnosis and treatment of lymph nodes.

## 5 Conclusion

In conclusion, the diagnostic timeliness, sensitivity, accuracy and consistency with pathological gold standard of AI-ROSE in CT-guided lymph node biopsy were significantly better than those of traditional exfoliated cytology. This technology is helpful to solve the clinical pain points of traditional ROSE, such as insufficient human resources, high missed diagnosis rate of conventional cytology, and delayed diagnosis. It is convenient to operate and time-sensitive, and has broad clinical application prospects. Under the premise of strict specimen preparation and staining quality control, AI-ROSE can be used as an efficient and rapid screening tool in lymph node biopsy, optimize the interventional diagnosis and treatment process, and provide strong support for the early diagnosis and individualized treatment of lymph node lesions.

## Data Availability

The original contributions presented in the study are included in the article/Supplementary Material, further inquiries can be directed to the corresponding author.
